# Sensory deafferentation modulates and redistributes neurocan in the rat auditory brainstem

**DOI:** 10.1002/brb3.1353

**Published:** 2019-07-04

**Authors:** Josef Heusinger, Heika Hildebrandt, Robert‐Benjamin Illing

**Affiliations:** ^1^ Neurobiological Research Laboratory, Department of Otorhinolaryngology University Medical Center Freiburg Germany

**Keywords:** auditory pathways, chondroitin sulfate proteoglycans, Gap43 protein, matrix metalloproteinase 2, regeneration

## Abstract

**Introduction:**

Cochlear ablation causing sensory deafferentation (*SD*) of the cochlear nucleus triggers complex re‐arrangements in the cellular and molecular communication networks of the adult mammalian central auditory system. Participation of the extracellular matrix (ECM) in these processes is not well understood.

**Methods:**

We investigated consequences of unilateral *SD* for the expression and distribution of the chondroitin sulfate proteoglycans, neurocan (Ncan) and aggrecan (Agg), alongside various plasticity markers in the auditory brainstem of the adult rat using immunohistochemical techniques.

**Results:**

In the deafferented ventral cochlear nucleus (VCN), Ncan expression increased massively within 3 postoperative days (POD), but rapidly decreased thereafter. Agg showed a similar but less pronounced progression. Decrease in Ncan was spatially and temporally related to the re‐innervation of VCN documented by the emergence of growth‐associated protein Gap43 contained in nerve fibers and presynaptic boutons. Concurrently, astrocytes grew and expressed matrix metalloproteinase‐2 (MMP2), an enzyme known to emerge only under re‐innervation of VCN. MMP2 is capable of cleaving both Ncan and Agg when released. A transient modulation of the ECM in the central inferior colliculus on the side opposite to *SD* occurred by POD1. Modulations of glutamatergic synapses and Gap43 expression were detected, reflecting state changes of the surrounding tissue induced by transsynaptic effects of *SD*.

**Conclusions:**

The ECM variously participates in adaptive responses to sudden deafness by *SD* on several levels along the central auditory pathway, with a striking spatial and temporal relationship of Ncan modulation to astrocytic activation and to synaptogenesis.

## 1 SIGNIFICANCE OUTCOMES

- The extracellular matrix is variously involved in brain plasticity, but the details of its regulation are still scantly known.
- This study investigates neurocan and aggrecan upon sensory deafferentation in the auditory brainstem, showing a fast rise of their expression followed by a rapid decay.
- These reactive changes are locally and temporally associated with astrocytic growth, astrocytic expression of MMP2, and the emergence and maturation of Gap43 containing presynaptic profiles.
- Understanding the regulation of extracellular matrix in brain plasticity may be useful for future treatments of brain injury, such as introduction of matrix‐cleaving enzymes.

## 2 INTRODUCTION

Although the extracellular matrix (ECM) in the brain, particularly enriched in so‐called perineuronal nets (PNNs), already caught the attention of Camillo Golgi and Santiago Ramón y Cajal in the 19th century (Celio, Spreafico, Biasi, & Vitellaro‐Zuccarello, 1998), its significance for nervous function was accepted not before later in the 20th century. Major components of PNNs are the chondroitin sulfate proteoglycans (CSPGs) of the lectican family, brevican, neurocan (Ncan), aggrecan (Agg), and versican. While ECM is rich in Ncan and versican isoforms V0 and V1 early in the ontogeny of mammalian central nervous systems (CNS), only small amounts of Agg, the most common CSPG in the adult brain, are found (Matthews et al., 2002; Milev et al., 1998; Oohira, Matsui, Watanabe, Kushima, & Maeda, 1994; Rauch et al., 1991). Beginning by about 2 weeks after birth, ECM is restructured. Much of Ncan and versican isoforms V0 and V1 disappear from the PNNs, while Agg expression increases through the following 5 months, reaching a plateau that is maintained throughout adulthood (Milev et al., 1998).

PNNs are present throughout the central auditory system (Sonntag, Blosa, Schmidt, Rübsamen, & Morawski, 2015). CSPG immunoreactivity reaches a peak in the pontine auditory nuclei by about postnatal day 12 (Friauf, 2000), the time of hearing onset in the rat (Blatchley, Cooper, & Coleman, 1987). As sensory deprivation leads to an impairment of PNNs, for example, around neurons of the superior olivary complex (SOC) in deaf rats (Myers, Ray, & Kulesza, 2012) or in the visual cortex of dark‐reared animals (Pizzorusso et al., 2002), sensory stimulation appears to be essential for proper PNN maturation. After termination of critical developmental periods, PNNs are thought to serve synaptic stabilization.

The stability of established PNNs can be affected by lesions to the CNS. CSPGs are upregulated in the glial scar around experimentally induced lesions in adult mammalian brains (Bovolenta, Wandosell, & Nieto‐Sampedro, 1993; Levine, 1994; McKeon, Schreiber, Rudge, & Silver, 1991). Under these circumstances, CSPGs are major inhibitors for axonal growth (Silver & Miller, 2004). Conversely, cleavage of CSPGs by experimental application of ChABC causes axonal sprouting (Song & Dityatev, 2018; Wang & Fawcett, 2012). Around brain lesions, major sources of CSPGs are reactive astrocytes (Asher et al., 2000; Haas, Rauch, Thon, Merten, & Deller, 1999; McKeon, Jurynec, & Buck, 1999). Astrocytes variously participate in lesion‐associated reorganization. They are involved in the cleanup of cellular debris after axon degeneration (Bechmann & Nitsch, 1997; Vaughn & Pease, 1970). Furthermore, they support nerve regeneration, synaptogenesis, and establishment of synaptic connectivity (Fredrich, Zeber, Hildebrandt, & Illing, 2013; Theodosis, Poulain, & Oliet, 2008). Synapse formation and maintenance are associated with astroglial‐derived ECM (Dityatev & Schachner, 2006; Faissner et al., 2010).

The motivation of this study was to investigate the involvement of ECM in the reorganization of the adult auditory brainstem, using a model of sudden and complete sensory deafferentation (*SD*) of central nervous networks. The central auditory system encompasses complex synaptic connectivity to support spatial hearing. The cochlear part of the 8th cranial nerve connects the cochlea with the equilateral cochlear nucleus from which auditory pathway splits into different ascending tracks. Three major fiber bundles are the ventral, intermediate, and dorsal acoustic striae. The ventral acoustic stria projects to the SOC bilaterally, both intermediate and dorsal acoustic striae decussate across the medulla, and fibers join the lateral lemniscus of the contralateral side. Thus, the inferior colliculus in the midbrain is dominated by input from the cochlea of the opposite side.

Cochlear ablation inflicts degeneration of the auditory nerve, leading to *SD* of the cochlear nucleus. This entails a massive reduction of excitatory glutamatergic input to the cochlear nucleus (Hildebrandt, Hoffmann, & Illing, 2011). This wrecking process is quickly accompanied by constructive activity involving functional re‐innervation of the cochlear nucleus by axon collaterals originating from medial olivocochlear neurons of the ventral nucleus of the trapezoid body (Kraus & Illing, 2004). These in‐growing fibers contain the growth‐associated protein (Gap43), a marker for axonal growth and synaptogenesis (Benowitz & Routtenberg, 1997).

We focused on the distribution of CSPGs in PNNs of several auditory brainstem regions, studying their reactive dynamic and their relation to the changing populations of synaptic contacts. Motivated by its abundance in juvenile but not mature brains, we focused on Ncan and compared it to Agg which is abundant in PNNs of adult brains. If the ECM specifically contributes to the dynamic maintenance of neural net function, it was expected to respond in specific local and temporal relation to other cellular and molecular events that are already known to be involved into the constructive reorganization of the auditory brainstem upon *SD*.

## 3 MATERIALS AND METHODS

### 3.1 Animals

This study is based on the brains of 77 mature Wistar rats aged 7–20 weeks (190–250 g; Charles River, RRID: RGD_13508588). For reasons of physiological stability under anesthesia, female rats were used. Care and use of the animals were approved by the appropriate agency (Regierungspräsidium, permission number G‐13/81 & G16/168). For cochlear ablation, rats were deeply anesthetized by inhaling isoflurane (Forene, AbbVie). Preceding surgery, rats received a subcutaneous injection of carprofen (Carprieve, Norbrook, 5 mg/kg body weight) for pain reduction, followed by daily oral application of carprofen (Rimadyl, Zoetis, 25 mg/kg body weight). Rats were divided into five experimental groups, surviving 1 (*n* = 12), 3 (*n* = 18), 4 (*n* = 1), 7 (*n* = 25), or 14 (*n* = 8) days after surgery, and a nonoperated control group (*n* = 13).

### 3.2 Sensory deafferentation

Tympanic membranes were checked for integrity and transparency bilaterally. Unilateral *SD* caused by cochlear ablation was invoked on the left side as described previously (Illing, Horváth, & Laszig, 1997). In short, the facial nerve was severed upon its exit from the skull. The bulla tympani was opened by removing tympanic membrane and stapes. The opening was widened for full visibility of the cochlea. The bony wall was perforated using a spherical drill, and the interior, including the spiral ganglion, was thoroughly cleared. Cochlea and middle ear cavity were subsequently filled with gel foam, and the skin was surgically closed. In the present study, *SD* was always done unilaterally. The lesioned and thus deafferented side will henceforth be referred to as ipsilateral.

### 3.3 Brain preparation

After a predetermined postoperative survival period, rats received a lethal dose of sodium thiopental (Thiopental Inresa; Inresa, i.p. 50 mg/ml per 200 g body weight). At respiratory arrest, they were transcardially perfused first with Tyrode's solution to empty the circulatory system of blood and then with a fixation solution containing 4% paraformaldehyde (PFA) and 0.025% glutaraldehyde in 0.1 M phosphate buffer (PB, pH 7.4) for 75 min. Subsequently, brains were removed from the skull and the operated side was marked by a longitudinal incision. For immunostaining with 3,3′‐diaminobenzidine tetrahydrochloride (DAB) and double or triple immunofluorescence staining, tissue was soaked overnight in 30% sucrose for cryoprotection at 4°C before being frozen and cut into 30‐µm‐thin frontal sections using a cryotome (CM3050 S, Leica, RRID: SCR_016844).

### 3.4 Immunohistochemistry

Free‐floating sections were washed in 50 mM glycine in 0.02 M phosphate‐buffered saline (PBS). For DAB staining, sections were treated with 0.1% Triton, 0.045% H_2_O_2_, 1% milk powder, and 5% normal serum, all in PBS at pH 7.4 for 30 min each, and thoroughly rinsing in PBS between steps. Sections were then incubated with primary antibodies (Table 1) for 36 hr at 4°C. For control sections, primary antibody was omitted to test for nonspecific binding of the secondary antibody. After rinsing in PBS, matching biotinylated secondary antibodies (Table 1) were applied and an avidin–biotin–peroxidase complex was allowed to form (Elite Vectastain ABC Kit; Vector; RRID: AB_2336819) for 30 min. Sections were once again washed in PBS and then transferred to 50 mM Tris buffer at pH 7.2, before being stained with 0.05% DAB (Sigma), 0.3% ammonium nickel sulfate, and 0.0015% H_2_O_2_ in Tris buffer. Before mounting on gelatin‐coated slides, sections were rinsed in Tris buffer followed by PBS. Mounted slides were air‐dried, dehydrated in alcohol and xylene, and coverslipped with Entellan (Merck).

**Table 1:** Antibodies used in the study

Antigen	Description of immunogen	Source, host species, catalog no., RRID	Dilution (DAB/FL/EM)
Primary antibodies			
Aggrecan	Mouse aggrecan, amino acids 1177–1326	Millipore, rabbit polyclonal, AB1031, AB_90460	1:2,000/−/−
Gad65	Purified rat brain glutamic acid decarboxylase, 65 kDa isoform	Millipore, mouse monoclonal, MAB351, AB_2263126	1:2,000/1:1,000/1:500
Gap43	Purified rat brain growth‐associated protein 43	Millipore, mouse monoclonal, MAB347, AB_94881	1:5,000/1:1,000/1:1,000
GFAP	Glial fibrillary acidic protein from pig spinal cord	Sigma‐Aldrich, mouse monoclonal, G3893, AB_477010	–/1:5,000/–
HuC/HuD	Human HuC/HuD neuronal protein	Molecular Probes, mouse monoclonal, A−21271, AB_221448	–/1:125/–
MMP2	Human MMP2, amino acids 1–76	Santa Cruz, rabbit polyclonal, sc−10736, AB_2250826	–/1:40/1:20
Neurocan	Mouse/rat neurocan, full‐length form and proteolytic fragments	R&D Systems, sheep polyclonal, AF5800, AB_2149717	1:1,000/1:500/1:500
vGluT1	Synthetic peptide from rat vesicular glutamate transporter 1	Millipore, guinea pig polyclonal, AB5905, AB_2301751	1:5,000/1:1,000/–
Secondary antibodies			
Guinea pig IgG biotinylated		Vector, goat, BA‐7000, AB_2336132	1:200
Mouse IgG biotinylated		Vector, horse, BA‐2001, AB_2336180	1:200
Rabbit IgG biotinylated		Vector, goat, BA‐1000, AB_2313606	1:200
Sheep IgG biotinylated		Vector, rabbit, BA‐6000, AB_2336217	1:200
Mouse IgG Alexa 405		Abcam, donkey, ab175659, (no RRID)	1:200
Sheep IgG Alexa 488		Molecular Probes, donkey, A‐11015, AB_141362	1:200
Guinea pig IgG Cy3		Millipore, donkey, AP193C, AB_92669	1:200
Mouse IgG Cy3		Millipore, donkey, AP192C, AB_92642	1:200
Rabbit IgG Cy3		Millipore, donkey, AP182C, AB_92588	1:200
Mouse FAB Nanogold		Nanoprobes, goat, 2002, AB_2637031	1:100
Rabbit FAB Nanogold		Nanoprobes, goat, 2004, (no RRID)	1:100

For immunofluorescence double and triple labeling, free‐floating sections were pre‐incubated with 0.1% Triton in PBS for 30 min, followed by washing in PBS and incubation of the primary antibodies (Table 1) at 4°C. In case of Gap43, we added 0.05% Triton to the medium. After 24 hr, primary antibodies were washed out with PBS and sections were incubated with normal donkey serum prior to the respective secondary antibodies (Table 1) labeled with fluorochromes. To test for cross‐reactivity, the first primary antibodies were combined with secondary antibodies, raised from the species of the second primary antibody. Following incubations with secondary and, if required, third primary antibody, each with the corresponding secondary antibody, sections were counterstained with 0.2% Sudan Black B (Carl Roth) in 70% ethanol to reduce background staining (Schnell, Staines, & Wessendorf, 1999) and mounted on gelatin‐coated slides. We proceeded with air‐drying overnight, followed by aqueous coverslipping with Mowiol 4‐88 (Carl Roth) containing 2.5% triethylenediamine (DABCO, Carl Roth) to stabilize fluorochromes.

### 3.5 Light microscopy

For DAB staining, photographs were obtained with a digital camera (AxioCam; Zeiss) attached to a light microscope (Zeiss Axiophot, objectives: ×100 oil/NA 1.3, ×10/NA 0.3, ×5/NA 0.15, acquisition software: AxioVision, RRID: SCR_002677) at eight‐bit gray tone depth.

For the evaluation of spatial affiliation of cellular elements specified by the presence of a specific antigen, fluorescence images were obtained using a laser scanning confocal microscope (Leica TCS SP8, Leica, objectives: ×63 oil/NA 1.4, ×20/NA 0.75, RRID: SCR_002140, acquisition software: Leica Application Suite X, RRID: SCR_013673) equipped with argon lasers and a UV diode for visualization of Alexa 405, Alexa 488, and Cy3. Settings of lasers and detectors were strictly held constant for each type of double labeling. Confocal stacks were postprocessed in Huygens Deconvolution (Scientific Volume Imaging B.V., RRID: SCR_014237). Overlay of the channels and image editing was performed with Fiji (Schindelin et al., 2012, RRID: SCR_002285). 3D animations shown in the supplementary material were done with Imaris (Ver. 9.2.0, Bitplane, RRID: SCR_007370).

### 3.6 Quantitative data acquisition

DAB images were used to perform statistical evaluation of the overall staining intensity of Ncan, Agg, Gap43, vesicular glutamate transporter 1 (vGluT1), and glutamate decarboxylase 65 (Gad65) in VCN and central inferior colliculus (CIC). Light microscopic images were taken with an ×10 objective of VCN and an ×5 objective of CIC. Settings of the light source and microscope were held strictly constant for the respective brain region. Images so obtained were globally processed in Photoshop (CS6 Ver.13, Adobe, RRID: SCR_014199). Rectangles of 250 × 250 pixels were defined as regions of interest (ROIs), centered in ipsilateral and contralateral VCN and CIC, respectively. Mean gray tones were determined in these ROIs. The values were then used to calculate the ipsilateral‐to‐contralateral ratio in VCN and the contralateral‐to‐ipsilateral ratio in CIC within each section. Per animal, we obtained 2–3 ratios in VCN and CIC, respectively. The mean ratio was calculated for VCN and for CIC of each animal. To determine whether change in ratios resulted from staining increases on one side or decreases in the other, across‐brain comparisons were made by measuring average gray values in contralateral VCN and ipsilateral CIC and respective sections from control brains.

### 3.7 Statistical evaluation

Gray tone ratios were determined, and across‐brain comparisons were done of controls and all survival groups using Prism 8.1.0 (GraphPad Software, Inc., RRID: SCR_002798). We tested our data for normal distributions (using the Kolmogorov–Smirnov and Shapiro–Wilk test) and equal variances (using Brown–Forsythe and Bartlett's test). Though our data were normally distributed, the assumption of homogeneity of variances was violated in several cases. Therefore, we turned to Welch's ANOVA to determine significant differences between the means of staining ratios in the respective groups, as this test is robust against heteroscedasticity (Levy, 1978). Multiple comparisons were submitted to Dunnett's T3 post hoc test. In the figures, data are presented as scatter dot plot with means connected by line. Significance was set to *p* < 0.05, and significance levels are indicated as ***p < 0.001, **p < 0.01, or *p < 0.05. Statistical results of Welch's ANOVA and Dunnett's T3 post hoc tests including means and standard error of means (SEMs) are presented in Table 2 for VCN and in Table 3 for CIC. In the figures, significance bars were only shown for the most relevant differences, with a complete listing provided in Tables 2 and 3.

### 3.8 Electron microscopy

Following perfusion and brain preparation as described above, brains were postfixed in 4% PFA for 20 hr at 4°C. They were cut into 30‐µm‐thin frontal sections on a vibratome (VT 1000S, Leica, RRID: SCR_016495) and collected in 50 mM glycine in PBS. Free‐floating sections were permeabilized for 10 min with 5% and 10% dimethyl sulfoxide and for 20 min with 20% and 40%. Following each step, sections were rinsed in PBS. Endogenous peroxidase activity was blocked with 0.045% H₂O₂ in PBS, and nonspecific binding sites were blocked with 5% goat serum in PBS each for 30 min. The first primary antibody (Table 1) was applied in PBS with 1% serum and 0.025% cold water fish skin (CWFS) gelatin and incubated for 72 hr at 4°C. Thereupon, a biotinylated anti‐sheep antibody (Table 1) in PBS was applied to the sections for 1 hr at room temperature (RT) with 5% goat serum for 30 min. The second primary antibody (Table 1) was incubated for 20 hr at 4°C. Subsequently, a FAB nanogold (Table 1) raised against the species of the second primary antibody was added for 20 hr at RT in PBS with 0.1% BSA‐c (Aurion) and 0.1% CWFS. Sections were postfixed for 10 min with 2% glutaraldehyde in PBS and washed with 100 mM glycine in PBS. The nanogold was enhanced with GoldEnhance EM (Nanoprobes) for 9 min, and the reaction was stopped by applying 3% sodium thiosulfate in aqua dest for 30 s. The sections were then incubated with the avidin–biotin–peroxidase complex (Elite Vectastain ABC Kit; see above) for 1 hr at RT in PBS. Binding sites of the first primary antibody were visualized by the DAB reaction (0.05% DAB, 0.0015% H₂O₂ in 50 mM Tris, pH7.2), incubating for 5 min. Sections were postfixed in 0.2% osmium tetroxide in H₂O, dehydrated in ethanol and propylene oxide, and flat‐embedded in EMbed 812 (Science Services). The central region of the anterior VCN was trimmed from the embedded sections, cut ultrathin at 100 nm, and without further staining examined under a Tecnai electron microscope (Fei). In ultrathin double‐labeled sections, DAB staining is recognizable by a darkening of immunoreactive structures whereas immunogold staining is characterized by small black dots.

## 4 RESULTS

### 4.1 *SD*‐dependent Ncan expression in the auditory brainstem

**Figure 1:**
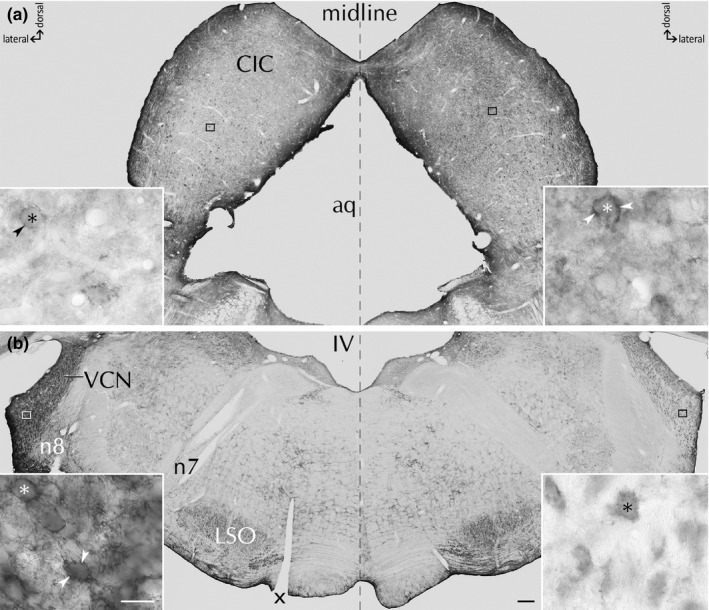
Ncan expression following *SD*. Ncan is shown in CIC by POD1 (a) and in VCN by POD3 (b). Insets show higher magnification of Ncan(+) PNNs (arrowheads). *SD* side is marked by incision (*x*). Neuronal cell bodies are indicated by asterisks. LSO, lateral superior olive; n7, facial nerve; n8, vestibulocochlear nerve; aq, cerebral aqueduct; IV, fourth ventricle. Scale bar for a, b: 200 µm, scale bar for insets: 20 µm

Upon unilateral *SD*, Ncan expression strongly increased in ipsilateral VCN and contralateral CIC (Figure 1).

**Table 2:** Statistical data of grayscale analysis in VCN

				Ncan		Agg		Gap43		Gad65		vGluT1	
VCN: staining ratio i/c	ANOVA overall		W (DFn,DFd)	23.29 (4, 15.91)		14.07 (4, 25.14)		27.04 (4, 19.85)		22.35 (4, 13.40)		20.16 (4, 12.60)	
			*p*‐value	<0.001***		<0.001***		<0.001***		<0.001***		<0.001***	
	No. of animals	mean ± *SEM*	Co	7	1.021 ± 0.007	6	0.986 ± 0.025	7	0.987 ± 0.007	5	0.993 ± 0.015	5	0.991 ± 0.016
			POD1	9	1.283 ± 0.081	10	1.112 ± 0.050	12	1.025 ± 0.009	9	1.074 ± 0.025	8	1.041 ± 0.023
			POD3	9	3.072 ± 0.336	10	1.427 ± 0.061	12	1.499 ± 0.139	6	1.202 ± 0.014	6	0.802 ± 0.022
			POD7	12	1.471 ± 0.075	12	1.196 ± 0.039	12	1.306 ± 0.036	7	1.123 ± 0.012	5	0.838 ± 0.043
			POD14	7	1.490 ± 0.099	7	1.303 ± 0.085	7	1.237 ± 0.045	6	1.154 ± 0.051	3	0.842 ± 0.019
	*p*‐value from Dunnett's T3 multiple comparison test		Co vs. POD1	0.089		0.299		0.035*		0.137		0,548	
			Co vs. POD3	0.003**		<0.001***		0.031*		<0.001***		<0.001***	
			Co vs. POD7	0.001**		0.003**		<0.001***		0.008**		0,127	
			Co vs. POD14	0.024*		0.067		0.011*		0.150		0,012*	
			POD1 vs POD3	0.005**		0.008**		0.049*		0.008**		<0.001***	
			POD1 vs. POD7	0.613		0.849		<0.001***		0.804		0.042*	
			POD1 vs. POD14	0.674		0.480		0.018*		0.801		0.001**	
			POD3 vs. POD7	0.010*		0.049*		0.840		0.119		0.994	
			POD3 vs. POD14	0.013*		0.912		0.559		0.972		0.8173	
			POD7 vs. POD14	>0.999		0.920		0.906		0.999		>0.9999	
VCN: across‐brain comparison of gray values on right with contralateral sides	ANOVA overall		W (DFn,DFd)	0.047 (4, 18.64)		2.057 (4, 17.71)		2.967 (4, 17.98)		2.485 (4, 12.80)		0.675 (4, 8.368)	
			*p*‐value	0.995		0.130		0.048*		0.096		0.627	
	No. of animals	mean ± *SEM*	Co	7	183.7 ± 4.471	6	145.5 ± 13.92	7	214.3 ± 6.697	5	205 ± 5.148	5	195.6 ± 9.464
			POD1	9	180.8 ± 10.06	10	111.3 ± 9.685	12	220.3 ± 2.301	9	213.4 ± 3.342	8	181.8 ± 7.345
			POD3	9	183.8 ± 5.193	10	121.2 ± 8.081	12	218.4 ± 1.967	6	211.5 ± 2.110	6	177.5 ± 4.145
			POD7	12	184.7 ± 6.999	12	136.4 ± 6.420	12	225.2 ± 1.014	7	219.1 ± 1.981	5	185.4 ± 12.25
			POD14	7	186.1 ± 7.056	7	137.9 ± 4.949	7	224.3 ± 2.447	6	216.5 ± 2.460	3	184.3 ± 9.387
	*p*‐value from Dunnett's T3 multiple comparison test		Co vs. POD1	>0.999		0.438		0.983		0.813		0.916	
			Co vs. POD3	>0.999		0.742		0.999		0.897		0.607	
			Co vs. POD7	>0.999		0.999		0.687		0.280		0.997	
			Co vs. POD14	>0.999		>0.999		0.801		0.470		0.980	
			POD1 vs. POD3	>0.999		0.994		0.999		>0.999		>0.999	
			POD1 vs. POD7	>0.999		0.334		0.474		0.771		>0.999	
			POD1 vs. POD14	>0.999		0.224		0.917		0.996		>0.999	
			POD3 vs. POD7	>0.999		0.769		0.067		0.174		0.998	
			POD3 vs. POD14	>0.999		0.583		0.513		0.723		0.993	
			POD7 vs. POD14	>0.999		>0.999		>0.999		0.988		>0.999	

Note

The upper half of the table shows statistic data for the ipsilateral‐to‐contralateral ratio in VCN for Ncan, Agg, Gap43, Gad65, and vGluT1. The lower half shows data of gray value comparisons in the right/contralateral VCN. Welch's ANOVA was used to determine whether means among groups differed significantly. Dunnett's T3 post hoc tests were applied. For each group, number of animals, mean, and *SEM* are indicated.

Abbreviations: W, W ratio of the Welch's ANOVA; DFn, degrees of freedom for numerator; DFd, degrees of freedom for denominator.

Significance levels are indicated as ***p < 0.001, **p < 0.01, or *p < 0.05.

**Table 3:** Statistical data of grayscale analysis in CIC

				Ncan		Agg		Gap43		Gad65		vGluT1	
CIC: staining ratio *c*/*i*	ANOVA overall		W (DFn,DFd)	6.158 (4, 18.41)		4.308 (4, 18.67)		4.018 (4, 19.59)		19.48 (4, 12.16)		6.025 (4, 8.435)	
			*p*‐value	0.003**		0.012*		0.015*		<0.001***		0.014*	
	No. of animals	mean ± *SEM*	Co	6	1.000 ± 0.022	6	0.973 ± 0.020	7	0.986 ± 0.025	5	1.001 ± 0.007	5	0.984 ± 0.012
			POD1	10	1.278 ± 0.068	10	1.116 ± 0.030	12	1.166 ± 0.036	9	1.059 ± 0.016	8	1.05 ± 0.009
			POD3	8	1.097 ± 0.047	10	1.046 ± 0.027	12	1.048 ± 0.019	6	0.861 ± 0.023	6	1.013 ± 0.023
			POD7	12	0.994 ± 0.015	11	0.980 ± 0.024	12	1.024 ± 0.016	7	0.882 ± 0.023	5	0.976 ± 0.035
			POD14	6	0.947 ± 0.020	7	1.022 ± 0.022	7	1.036 ± 0.027	5	0.900 ± 0.020	3	0.973 ± 0.017
	*p*‐value from Dunnett's T3 multiple comparison test		Co vs. POD1	0.022*		0.013*		0.008**		0.074		0.013*	
			Co vs. POD3	0.520		0.351		0.455		0.009**		0.933	
			Co vs. POD7	>0.999		>0.999		0.871		0.013*		>0.999	
			Co vs. POD14	0.563		0.669		0.826		0.036*		0.999	
			POD1 vs. POD3	0.319		0.593		0.089		<0.001***		0.772	
			POD1 vs. POD7	0.019*		0.023*		0.025*		0.001***		0.465	
			POD1 vs. POD14	0.006**		0.197		0.090		0.002**		0.130	
			POD3 vs. POD7	0.410		0.552		0.976		0.998		0.980	
			POD3 vs. POD14	0.124		0.998		>0.999		0.877		0.816	
			POD7 vs. POD14	0.525		0.877		>0.999		0.999		>0.999	
CIC: across‐brain comparison of gray values on left with ipsilateral sides	ANOVA overall		*W (DFn,DFd)*	1.853 (4, 15.90)		1.711 (4, 16.54)		1.768 (4, 18.94)		3.734 (4, 11.75)		0.087 (4, 9.568)	
			*p*‐value	0.168		0.195		0.177		0.035*		0.985	
	No. of animals	mean ± *SEM*	Co	6	153.7 ± 6.515	6	91.7 ± 15.08	7	159.1 ± 12.85	5	145.6 ± 11.84	5	182.6 ± 12.29
			POD1	10	177.7 ± 11.45	10	90.3 ± 10.98	12	137.3 ± 10.56	9	172.6 ± 11.88	8	186 ± 11.08
			POD3	8	166 ± 7.201	10	90.7 ± 5.762	12	127.4 ± 6.057	6	134 ± 9.022	6	181.5 ± 6.941
			POD7	12	176.8 ± 5.390	11	112.4 ± 6.234	12	148.2 ± 7.771	7	171 ± 4.884	5	190.6 ± 13.39
			POD14	6	164 ± 14.29	7	94.6 ± 10.79	7	130.1 ± 12.78	5	147.2 ± 10.43	3	183.3 ± 7.535
	*p*‐value from Dunnett's T3 multiple comparison test		Co vs. POD1	0.542		>0.999		0.854		0.683		>0.999	
			Co vs. POD3	0.875		>0.999		0.337		0.991		>0.999	
			Co vs. POD7	0.144		0.864		0.995		0.499		>0.999	
			Co vs. POD14	0.997		>0.999		0.687		>0.999		>0.999	
			POD1 vs. POD3	0.987		>0.999		0.993		0.177		>0.999	
			POD1 vs. POD7	>0.999		0.590		0.991		>0.999		>0.999	
			POD1 vs. POD14	0.995		>0.999		>0.999		0.685		>0.999	
			POD3 vs. POD7	0.909		0.162		0.351		0.054		0.998	
			POD3 vs. POD14	>0.999		>0.999		>0.999		0.968		>0.999	
			POD7 vs. POD14	0.984		0.791		0.899		0.446		>0.999	

Note

The upper half of the table shows statistic data for the contralateral‐to‐ipsilateral ratio in CIC for Ncan, Agg, Gap43, Gad65, and vGluT1. The lower half shows data of gray value comparisons in the left/ipsilateral CIC. Welch's ANOVA was used to determine whether means among groups differed significantly. Dunnett's T3 post hoc tests were applied. For each group, number of animals, mean, and *SEM* are indicated.

Abbreviations: W, W ratio of the Welch's ANOVA; DFn, degrees of freedom for numerator; DFd, degrees of freedom for denominator.

Significance levels are indicated as ***p < 0.001, **p < 0.01, or *p < 0.05.

### 4.2 Modulation of Ncan Expression in VCN

**Figure 2:**
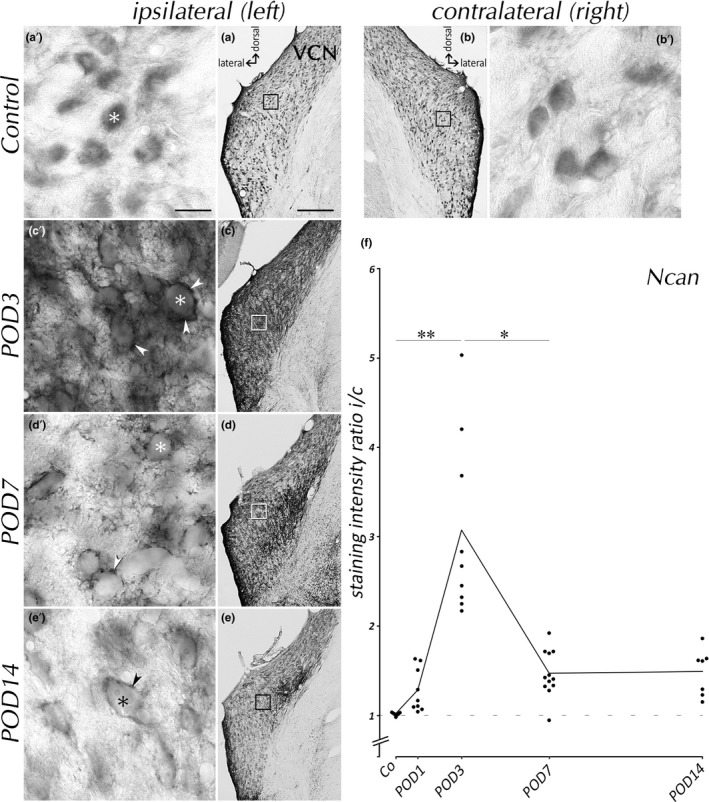
Time course of Ncan expression in VCN. Ncan transiently rose in ipsilateral VCN with a maximum around POD3 (c), followed by a decline toward POD7 (d) and POD14 (e). Higher magnification (a′–e′) reveals Ncan(+) PNNs (arrowheads) by POD3, POD7, and POD14 but not in controls. Neuronal cell bodies are indicated by asterisks. Scale bar for a–e: 200 µm, scale bar for a′–e′: 20 µm. (f) Analysis of ipsilateral(i)‐to‐contralateral(c) staining ratio, indicating a significant rise of Ncan by POD3 and its subsequent decline. Means are connected by line. Dashed line indicates symmetry between ipsilateral and contralateral sides

Ncan expression was determined before and after *SD* (Figure 2). In control animals, Ncan staining in VCN was predominantly found in neuronal cell bodies (Figure 2a′ and b′). This situation changed drastically as a consequence of *SD*. By postoperative day 3 (POD3), Ncan expression was massively increased in the entire VCN on the ipsilateral side (Figure 2c). High‐power images from the center of the *SD*‐affected VCN show Ncan staining by POD3 localized in PNN surrounding cell bodies (Figure 2c′, arrowheads). Moreover, the pericellular space was enriched in Ncan. By POD7, Ncan expression was reduced and fragmented, leaving a darkly stained region medially adjacent to the vestibulocochlear nerve surrounded by tissue that was less intensely stained (Figure 2d). At higher magnification, Ncan seemed largely removed from cellular surfaces and the neuropil (Figure 2d′). Coverage of cell body surfaces was now disrupted (Figure 2d′, arrowhead). By POD14, Ncan was largely removed from VCN, with faint staining of PNNs still apparent ipsilaterally (Figure 2e′, arrowhead). No changes in Ncan expression were seen on the side contralateral to *SD* at any time after *SD*. Observations in VCN were equally applied for anteroventral (AVCN) and posteroventral cochlear nucleus (PVCN).

Staining intensities of Ncan were measured as gray values and plotted as left‐to‐right (controls) or ipsilateral‐to‐contralateral ratio of staining intensities (Figure 2f), on which statistical analyses are based (Table 2). Ncan ratio increased significantly by POD3, followed by a rapid decrease toward POD7. However, the staining ratios by POD7 and POD14 were still above controls.

To determine whether the changes of ratios were due to an ipsilateral increase or a contralateral decrease in staining intensity, gray tones were compared between controls to those in contralateral VCN of *SD* rats (Table 2, across‐brain comparison). Dunnett's T3 test revealed no statistically significant changes between the groups for Ncan and all other markers that will be introduced in the following. Obviously then, *SD*‐dependent changes of expression in the VCN occurred ipsilaterally rather than contralaterally. Changes in staining ratios can therefore be read as changes in ipsilateral staining for all molecular markers.

### 4.3 Modulation of Agg expression

**Figure 3:**
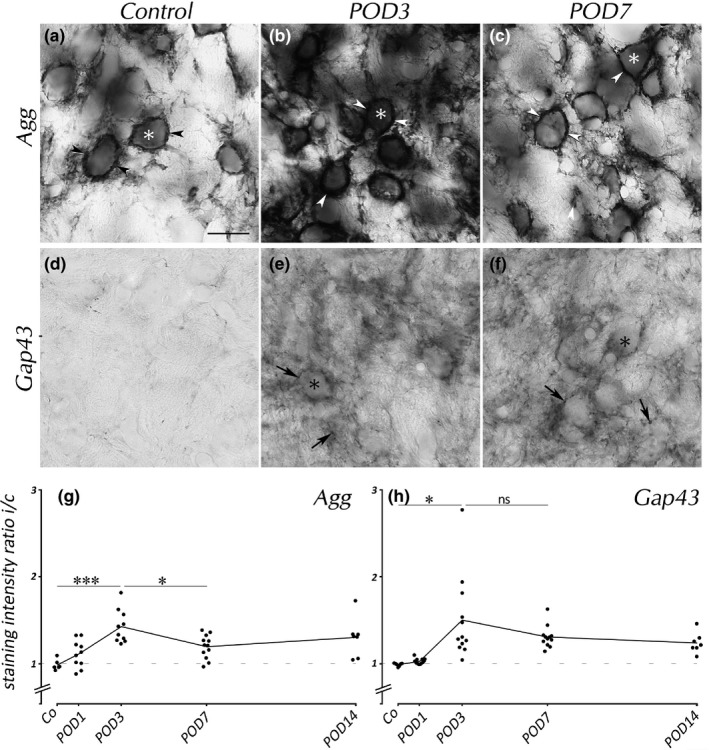
Agg and Gap43 in VCN. Agg(+) PNNs were present in controls (a, arrowheads). Their staining intensity rose by POD3 (b) and decreased toward POD7 (c). Gap43 immunoreactivity was hardly present in untreated adult brains (d) but rose toward POD3 (e). By POD3 and POD7, Gap43 mostly stained presynaptic boutons (arrows). Neuronal cell bodies are indicated by asterisks. Scale bar for a–f: 20 µm. Staining ratio of Agg (g) rose toward POD3 and declined thereafter. Gap43 staining ratio (h) was increased by POD3 and remained high by POD14

Expression of Agg in VCN upon *SD* (Figure 3a–c) showed dense staining of PNNs in controls (Figure 3a). *SD* increased expression by POD3 (Figure 3b), a progression that was reversed such that by POD7 (Figure 3c) PNNs resembled those in controls.

Statistics of Agg staining ratio (Figure 3g) in the VCN disclosed similar effects as for Ncan. It rose by POD3, followed by a significant decline toward POD7. However, compared to Ncan, peaks of Agg staining were distinctly smaller.

### 4.4 Temporal relation to plastic processes

These changes of ECM volume and composition were related to the concurrent *SD*‐dependent synaptogenesis shown by Gap43 staining (Hildebrandt et al., 2011; Illing et al., 1997). In controls, Gap43 staining was sparse (Figure 3d). By POD3, Gap43 expression was prominent in VCN (Figure 3e). Many presynaptic boutons containing Gap43 were arranged around unstained cell bodies (Figure 3e, arrows). A similar situation was encountered by POD7 (Figure 3f).

The analysis of staining ratios of Gap43 in the VCN (Figure 3h) revealed an increase in ipsilateral staining intensity by POD3. Unlike Ncan and Agg, there was no significant difference in staining ratio between POD3 and POD7 and between POD3 and POD14, indicating that the processes of axon growth and synaptogenesis were ongoing by 14 days after *SD*.

### 4.5 Spatial relation of Ncan to synaptic populations

**Figure 4:**
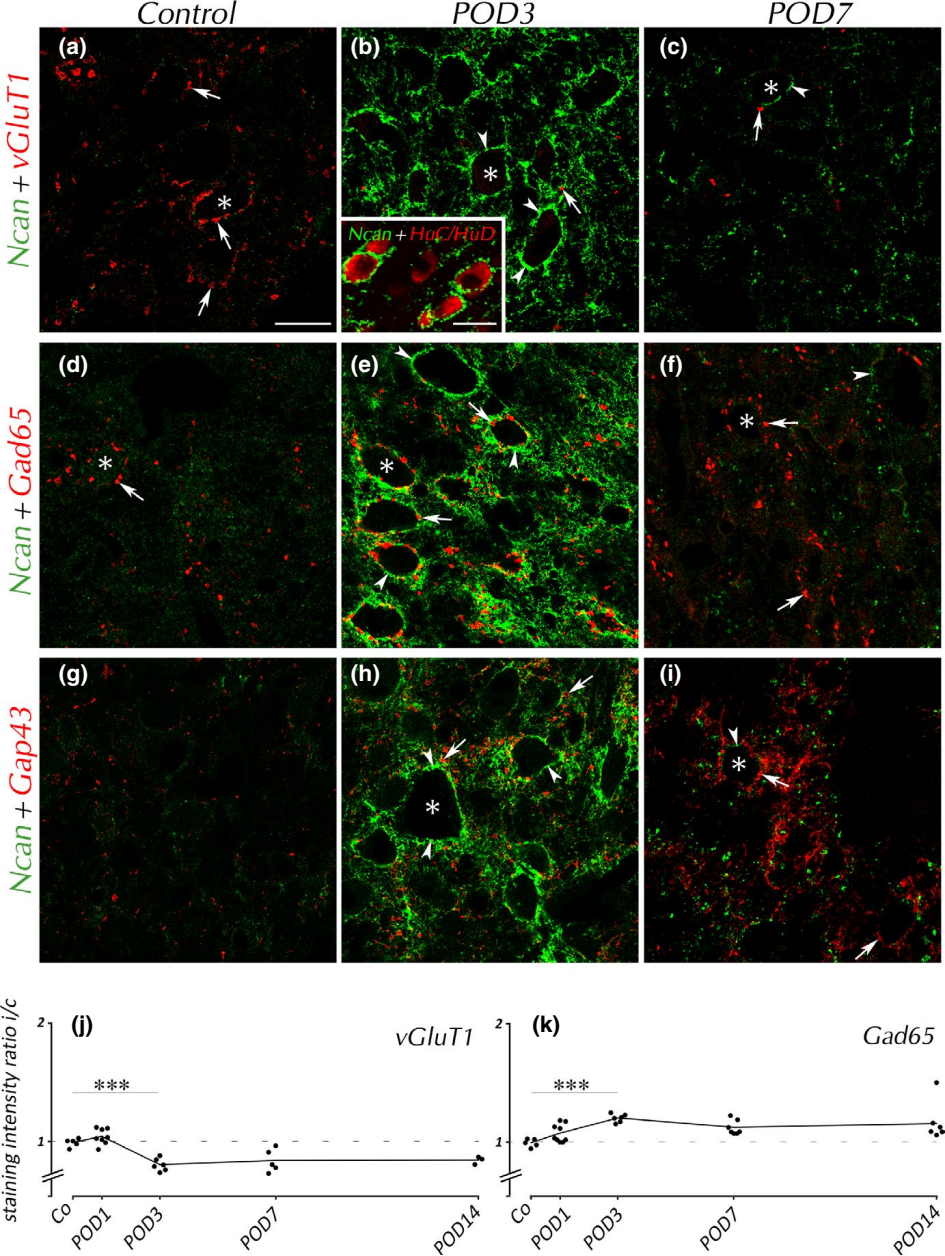
Ncan and synaptic markers in VCN. Changes in Ncan expression were accompanied by loss of glutamatergic synaptic boutons (a–c, arrows). Inset shows double labeling of Ncan and the neuronal marker HuC/HuD in VCN by POD3, with Ncan(+) PNNs exclusively covering neurons. GABAergic boutons (arrows) gained in staining intensity by POD3 (e) and POD7 (f). Most GABAergic boutons seemed to be embedded in Ncan(+) PNNs (arrowheads) by POD3 (e). Gap43(+) profiles (arrows) were often found close but outside the Ncan(+) PNNs by POD3 (h), but in contact to cell bodies by POD7 (i). Neuronal cell bodies are indicated by asterisks. Scale bar for a–i: 20 µm; scale bar for inset: 20 µm. In the quantification of vGluT1 (j), staining ratio dropped by POD3 and remained low. Ratio of Gad65 (k) rose by POD3

Aiming to define the effect of the temporal modulation of PNNs on different populations of mature and immature presynaptic boutons, we identified populations of excitatory and inhibitory synaptic contacts with antibodies raised against vGluT1 and Gad65, respectively. We observed *SD*‐dependent deterioration of glutamatergic boutons on the surface of VCN neurons (Figure 4a–c, arrows). In controls, boutons stained for vGluT1 were numerous and spread throughout VCN, and Ncan expression was marginal. By POD3, Ncan contained in PNNs was abundant (Figure 4b, arrowheads). By that time, most vGluT1(+) boutons have disappeared. Ncan disappeared thereafter, with only few glutamatergic endings remaining.

Findings for vGluT1 were confirmed in analysis of ipsilateral‐to‐contralateral ratio of gray values obtained from DAB images (Figure 4j). Ipsilateral staining decreased by POD3 and remained low henceforth.

GABAergic boutons stained by Gad65 (Figure 4d–f, arrows) were found pre‐ and postoperatively alike. These profiles tended to keep contact to cell bodies when emerging Ncan covered their surfaces (Figure 4e). Gad65 staining has turned somewhat brighter by POD3 (Figure 4e) and remained unaltered by POD7 when Ncan had almost vanished (Figure 4f). The ipsilateral‐to‐contralateral ratio of Gad65 staining rose by POD3 and stood high toward POD7 and POD14 (Figure 4k).

The emergence of a dense network of Gap43(+) fibers and boutons in the ipsilateral VCN by POD3 (Figure 4h, arrows) occurred concurrently with the rise of Ncan in PNNs. Unlike Gad65(+) boutons (Figure 4e), many Gap43(+) boutons seemed to reside distal to cell bodies outside the Ncan‐stained PNNs by POD3 (Figure 4h). By POD7, the population of Gap43(+) boutons has sustained while Ncan was hardly detectable anymore (Figure 4i). Now, Gap43(+) profiles seemed to be in contact with cell bodies.

### 4.6 Upregulation of MMP2 in reactive astrocytes

**Figure 5:**
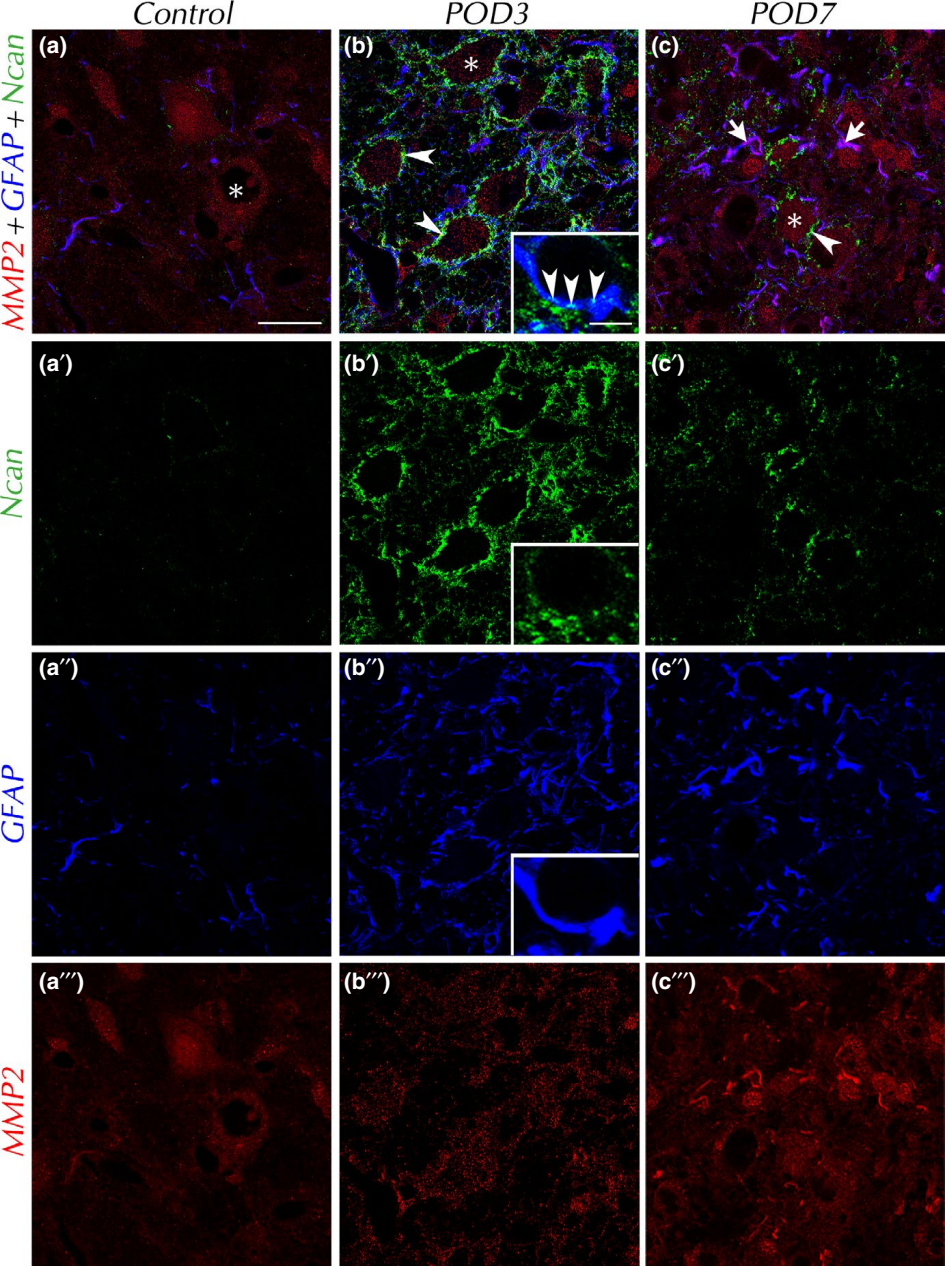
Fluorescence triple labeling of Ncan, matrix metalloproteinase‐2 (MMP2), and glial fibrillary acidic protein (GFAP) in VCN. By POD3, Ncan(+) PNNs (b, arrowheads) were touched by numerous activated GFAP(+) astrocytic processes. Inset shows high magnification of Ncan inside GFAP‐stained astrocyte. Concurrently to the decrease in Ncan toward POD7, MMP2 has risen in thick astrocytic fibers (c'''), while it was mainly found in neuronal somata and the neuropil in controls (a''') and by POD3 (b'''). Neuronal cell bodies are indicated by asterisks. Scale bar for a–c: 20 µm; scale bar for inset: 5 µm

Asking for the source of Ncan for its *SD*‐induced emergence and the reason of quick Ncan degradation shortly thereafter, the spatial and temporal expression of Ncan, matrix metalloproteinase‐2 (MMP2), and glial fibrillary acidic protein (GFAP) were put into relation (Figure 5). In VCN of control brains, MMP2 staining was weak and largely confined to neuronal cell bodies but could also be found in the neuropil. Astrocytic fibers were rare and only faintly stained for MMP2. By POD3, although astrocytes were now massively activated and their processes thick and numerous (Fredrich et al., 2013), levels of MMP2 were not obviously altered. GFAP often appeared in close vicinity to the now prominently present Ncan(+) PNNs. Frequently, we found Ncan staining within GFAP(+) profiles (Figure 5b inset; Supporting information). By POD7, MMP2 was seen to be enriched and redistributed, now often localized within GFAP(+) profiles (Figure 5c, arrows). This indicated an upregulation of MMP2 in astrocytes. By that time, Ncan has almost disappeared.

### 4.7 Glial scar in facial nucleus

Before lesioning the spiral ganglion, the facial nerve was severed during the procedure of cochleotomy. Similar to ipsilateral VCN, enhanced GFAP expression was seen by POD3 in the affected facial nucleus compared to the unoperated side (see Supporting information). Importantly, no changes were detected in the expression of Ncan or Agg due to the lesion of the facial nerve.

### 4.8 Electron microscopy of VCN

**Figure 6:**
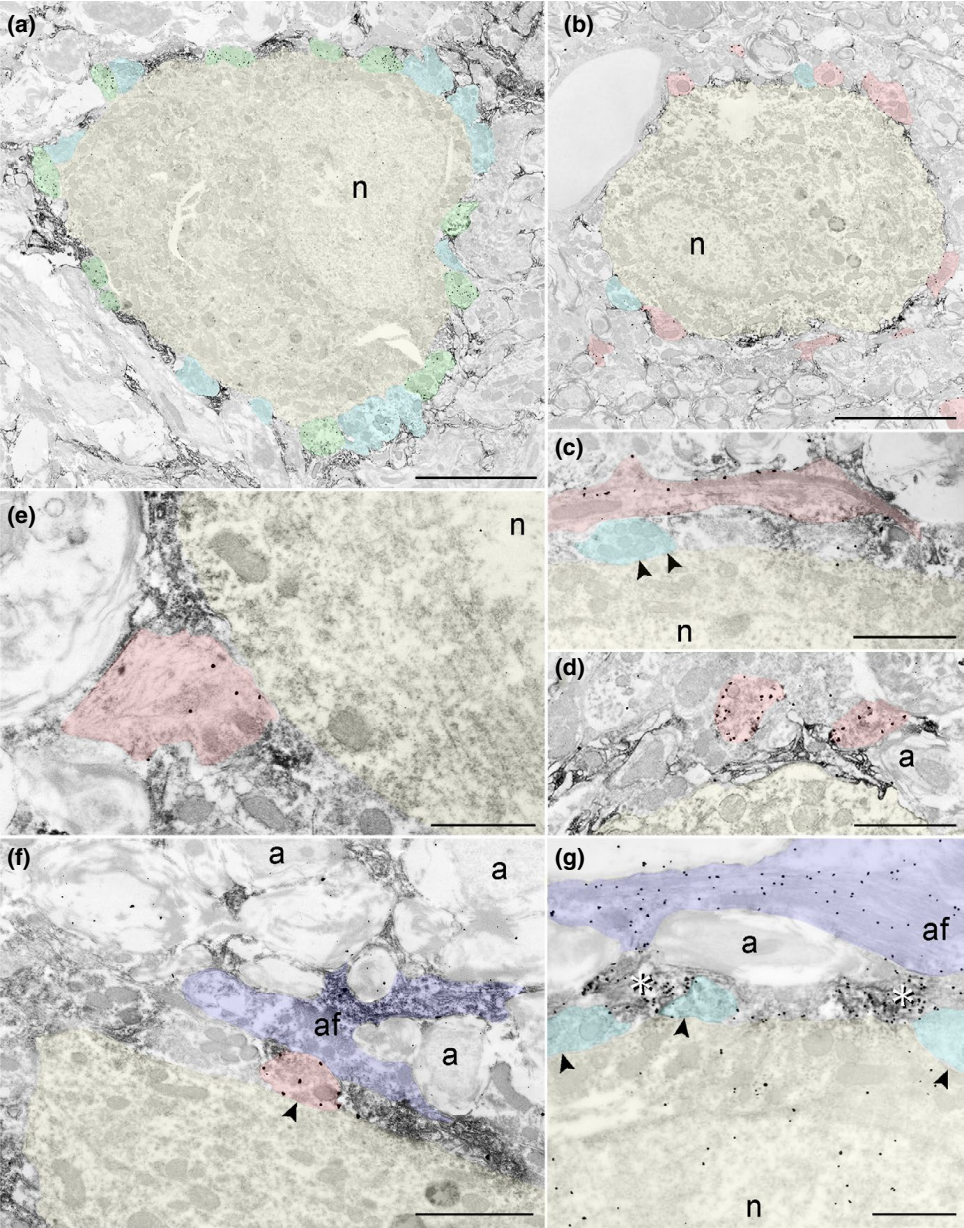
Ultrastructure of the deafferented VCN. GABAergic boutons (a, black dots, green profiles) and Gap43(+) boutons (b, black dots, red profiles) with DAB‐stained Ncan (darkened regions) are shown on or near neuronal somata by POD3, with Gap43(+) profiles laying in varying distances to the neuron (yellow profiles). Unstained synaptic boutons are marked as blue profiles. By POD4, Gap43(+) presynaptic profiles are found in different maturational states: a Gap43‐bearing fiber in a region darkly stained by Ncan (c), two immature Gap43(+) synapses distant to a neuron, separated from it by Ncan (d), a Gap43(+) growth cone (e) failing to show an active zone growing into the Ncan(+) PNN, and a mature Gap43(+) bouton (f) attached to a neuronal soma, forming a synaptic contact with the neuron. An astrocyte (af, purple profiles), residing in direct vicinity to a synaptic bouton, was found to be enriched with Ncan (dark staining). (g) Staining for Ncan (dark staining) and MMP2 (black dots) at POD4. MMP2 was found in astrocytes (purple) and neurons (yellow). In the neuropil, MMP2 colocated strongly with Ncan (asterisks). Postsynaptic densities marked by arrowheads; a, axon; af, astrocytic filaments; *n*, nucleus. Scale bar for a, b: 5 µm; scale bar for c, d, f: 2 µm; scale bar for e, g: 1 µm

For a better judgement on the spatial relations among cellular components and their molecular content, we investigated VCN tissue after *SD* on the ultrastructural level. Shortly after *SD* (POD3), GABAergic presynaptic endings (Figure 6a, green) showed an immediate proximity to neuronal cell bodies (yellow) partly embedded by Ncan identified by dark immunoreactivity on the *SD*‐affected side. By comparison, emerging Gap43(+) profiles (Figure 6b, red) were found in varying distance to the neuronal cell bodies at this time. Few of them were seen to touch upon a postsynaptic profile with a well‐defined synaptic cleft. By POD4 (Figure 6c–f), Gap43(+) profiles were present in regions of dense Ncan staining. A Gap43(+) fiber can be seen to have grown into a region rich in Ncan (Figure 6c). Two Gap43(+) synaptic boutons are shown near a Ncan‐covered neuron without forming a mature synaptic contact (Figure 6d) with the PNN still intact. These synaptic profiles are unattached to a plasma membrane and look morphological immature (Hildebrandt et al., 2011), suggesting that they have not reached their target and have not completed maturation. Another Gap43(+) growth cone (Figure 6e) has entered Ncan(+) PNN and contacted a cell body, lacking a visible active zone. A mature Gap43(+) synapse is shown in Figure 6f forming a mature synaptic contact (arrowhead) with the neuronal soma. This synaptic bouton neighbored to an astrocyte (purple) which contained noticeable amounts of Ncan. By POD4, MMP2 expression was mostly found in neurons, astrocytes, and neuropil (Figure 6g) where it was prominently present in neuropil regions rich in Ncan (asterisks), thought to begin Ncan degradation.

### 4.9 Changes in CIC

**Figure 7:**
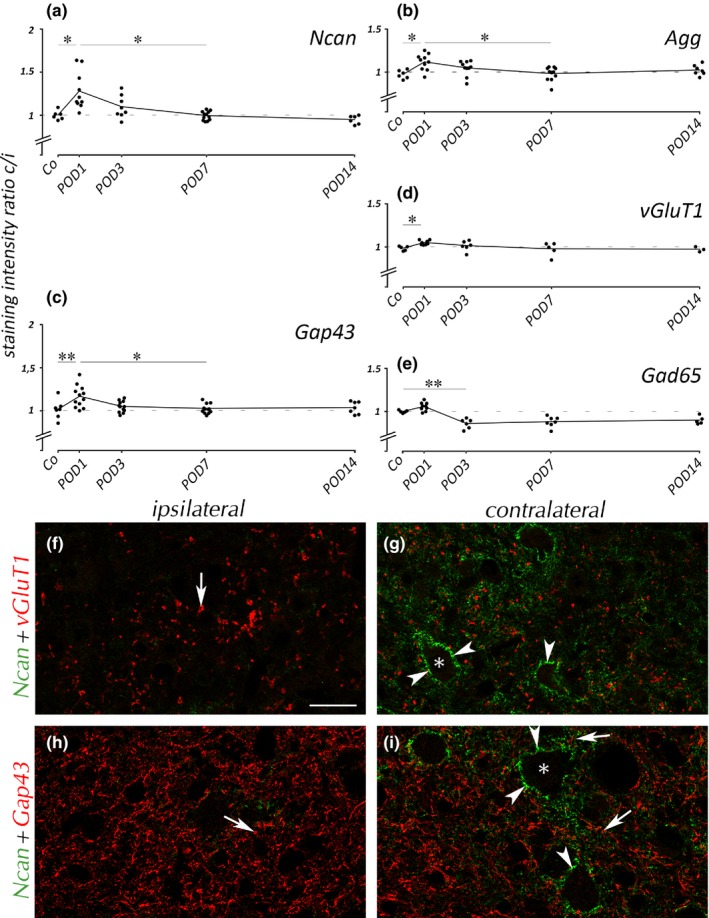
Effects in CIC. Graphs (a–e) show analysis of contralateral(c)‐to‐ipsilateral(i) staining ratio of mean gray values in CIC for all survival groups and 5 molecular markers. Means are connected by line. Dashed lines indicate symmetry between ipsilateral and contralateral sides. Ncan (a), Agg (b), Gap43 (c), and vGluT1 (d) all showed transient peaks of ratios by POD1. Only Gad65 (**e**) failed to show an effect by POD1 but decreased by POD3 to remain low. In fluorescence double labeling at POD1 in the CIC (f–i), Ncan staining dominated contralaterally and was mostly localized in PNNs (g, f, arrowheads). Staining differences for the synaptic markers vGluT1 and Gap43 were too subtle to be discernible in selected photographs but emerged by sampling from DAB‐stained sections (c, d). Synaptic boutons are indicated by arrows. Neuronal cell bodies are indicated by asterisk. Scale bar for f–i: 20 µm

When VCN was deafferented by cochlear ablation, transsynaptic effects developed in CIC. A significant change in Ncan staining was detected by POD1 (Figure 7a). By that time, modulations of expression were also observed for other molecular markers here employed (Figure 7b–d). Statistical data are shown in Table 3. DAB images of the ipsilateral CICs were taken from sections of control brains and all survival groups, normalized for background brightness, and submitted to statistical analysis (Table 3, across‐brain comparison). Dunnett's T3 post hoc test revealed no significant differences, indicating that the changes observed are due to the modulation of staining contralaterally, that is, on the side primarily affected by *SD* due to decussation of auditory pathways, rather than ipsilaterally.

Contralateral‐to‐ipsilateral staining ratios for Ncan (Figure 7 a) as well as for Agg (Figure 7 b), Gap43 (Figure 7 c), and vGluT1 (Figure 7 d) showed a rise of contralateral staining intensity by POD1. For all markers, this effect was neutralized by POD7 at the latest. This indicates a fast and transient subtle but significant transsynaptic response of the contralateral CIC to *SD* of VCN. Notably, this response precedes the molecular modulations encountered upstream in VCN.

Looking closer into the brief increase in staining intensity of Ncan in CIC contralateral to *SD* by POD1 (Figure 7f–i), we found that well‐defined PNNs have emerged by POD1 that was absent before. Bilateral differences in staining for vGluT1 and Gap43 by POD1 detected by averaging of gray values (Figure 7c,d) are too subtle in photographs of fluorescent staining to be appreciable. Conspicuously, Gap43 is present in a population of well‐defined boutons in CIC of the adult rat independent of *SD*. Gap43 was also found in CIC of control animals, which was suggested to imply conserved readiness for plastic dynamics rather than active axonal growth.

Gad65 expression (Figure 7 e) was modulated differently. There was no significant change in staining ratio between control and POD1, but a decrease has occurred by POD3. This asymmetry continued to POD7 and POD14. It seems to indicate that GABAergic inhibition in signaling networks of the contralateral CIC is persistently weakened due to *SD*‐induced deafness.

### 4.10 NCAN in DCN, LSO, and MGB

**Figure 8:**
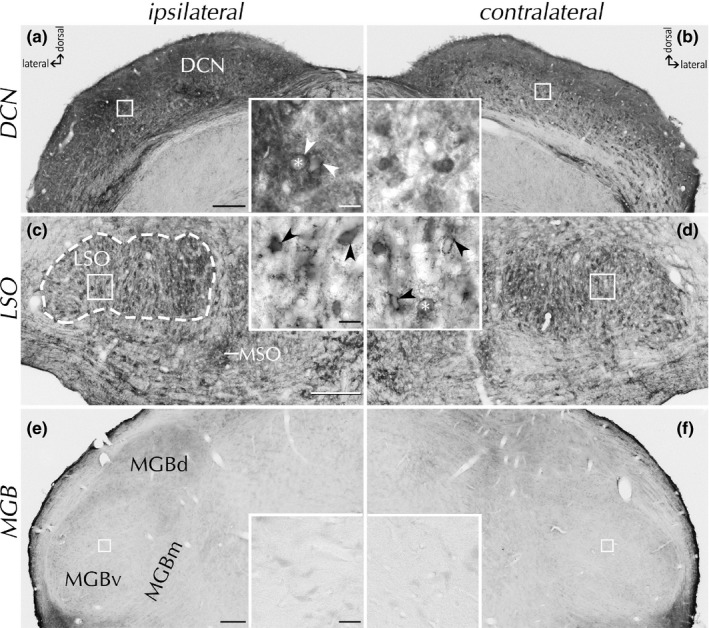
Ncan staining in dorsal cochlear nucleus (DCN), lateral superior olive (LSO), and medial geniculate body (MGB) by POD3. In DCN (a, b), a rise of Ncan expression pattern was reminiscent to that in VCN but weaker. In LSO (c, d, outlined by dashed line), we found scattered Ncan(+) PNNs (arrowheads) in controls and at all survival times, with no differences between hemispheres. In MGB (e, f) Ncan staining was absent from either side in any experimental group. Neuronal cell bodies are indicated by asterisk. MGBv, ventral part of the medial geniculate body; MGBd, dorsal part of the medial geniculate body; MGBm, medial part of the medial geniculate body; MSO, medial superior olive. Scale bars for a–d: 200 µm; scale bars for insets: 20 µm

Compared to VCN, the texture of Ncan staining in the dorsal cochlear nucleus (DCN) was different (Figure 8a,b). However, the *SD*‐induced temporal progression with an ipsilateral upregulation of Ncan in PNNs (arrowheads) by POD3 and a subsequent decline by POD7 was similar, although much less pronounced.

Within the superior olivary complex, differences in Ncan staining between the ipsilateral and contralateral side were not noted at any survival time (Figure 8c,d). Images at higher magnification (insets) show the presence of Ncan in PNNs (arrowheads) independent of *SD*.

We failed to find definite staining for Ncan in the MGB in control brains, and this situation did not change at any time after *SD* on either side (Figure 8e,f). However, PNNs were recognizable by their contents of Agg (Sonntag et al., 2015). Dense Agg staining was present in ventral MGB, but PNNs were hardly prominent dorsally. As for Ncan, Agg expression in the MGB remained unaffected by *SD*.

## 5 DISCUSSION

The major findings of this study are as follows: (a) Following *SD*, Ncan expression rises sharply and declines quickly; (b) reactive astrocytes contribute to the accumulation of Ncan; (c) the decline of Ncan expression correlates temporally to the emergence of MMP2 in astrocytes; (d) complex re‐arrangements of different synaptic populations are spatially and temporally associated with the modulation and redistribution of Ncan; and 5) a distinct but brief transsynaptic effect of *SD* manifests in the contralateral CIC, involving ECM and synaptic networks containing glutamate and Gap43, respectively.

### 5.1 Lesion‐dependent NCAN expression

Upregulation of Ncan expression in the adult brain as a consequence of lesions to the CNS has been reported before. It was observed after traumatic injuries of neocortex (Asher et al., 2000; McKeon et al., 1999), entorhinal cortex (Haas et al., 1999), the nigrostriatal pathway (Moon, Asher, Rhodes, & Fawcett, 2002), the spinal cord (Jones, Margolis, & Tuszynski, 2003; Tang, Davies, & Davies, 2003), and after kainate‐induced seizures (Matsui et al., 2002) or focal ischemia (Deguchi et al., 2005). Full‐length Ncan is found in the injured tissue (Asher et al., 2000), which is also recognized by the antibody used in the present study (Bekku & Oohashi, 2010). Whereas this form is abundant during development, only fragments of Ncan are found in the healthy adult brain (Matsui, Watanabe, & Oohira, 1994; Meyer‐Puttlitz et al., 1995; Rauch et al., 1991). Apparently, neuro‐ and glioplastic changes induced by brain injuries like the ones seen in VCN after *SD* re‐invoke Ncan expression reminiscent of ontogenetic dynamics.

### 5.2 Reactive astrocytes synthetized NCAN

During maturation of the mammalian CNS, Ncan is predominantly of neuronal origin (Engel, Maurel, Margolis, & Margolis 1996). By contrast, a lesion‐dependent rise of Ncan‐mRNA and Ncan‐protein is almost exclusively generated by reactive astrocytes (Asher et al., 2000; Deguchi et al., 2005; Haas et al., 1999; Jones et al., 2003; Matsui et al., 2002; McKeon et al., 1999). Our results are compatible with these reports as Ncan was found inside as well as immediately around astrocytes recognized by GFAP (light microscopy: Figure 5b inset; electron microscopy: Figure 6f; Supporting information). Following *SD*, GFAP intensity rises quickly and massively in VCN (Fredrich et al., 2013; Janz & Illing, 2014), indicating activation of these cells. Colocalization of Ncan and GFAP was most prominent by POD3, but still recognizable by POD7. We found no evidence for other than astrocytic origin of Ncan.

### 5.3 Following *SD*, NCAN rapidly decreased between POD3 and POD7 in VCN

After a peak of deafferentation‐dependent Ncan expression in VCN was reached by POD3, it declined over the following days, leveling at a plateau by POD7 maintained up to POD14 slightly above control level. PNNs stained for Ncan were found to be porous by POD7 and POD14. None of the aforementioned studies concerning a lesion‐dependent rise of Ncan immunoreactivity describes a comparably fast and near‐complete downregulation.

### 5.4 Ncan modulation corresponded spatially and temporally to re‐innervation

An outstanding aspect of *SD* by cochlear ablation is the fast, synchronous, and complete loss of sensory axons (Gentschev & Sotelo, 1973), followed by a broad synaptogenesis in the deafferented VCN (Hildebrandt et al., 2011; Illing et al., 1997). This re‐innervation, visualized by Gap43 immunoreactivity, becomes prominent by POD3. A previous study (Kraus & Illing, 2004) revealed that this re‐innervation depends on axon collaterals originating from medial olivocochlear neurons in the ventral nucleus of the trapezoid body. While staining for Gap43 sustained for the following days, Agg and in particular Ncan sharply declined. By POD3, fluorescence double staining revealed a local encounter of GAP43 and Ncan (Figure 4h), with Ncan still separating Gap43(+) profiles reminiscent of nascent presynaptic endings and the plasma membrane of a nearby neuron. By the same time, mature Gad65(+) synaptic contacts resided directly on these neuronal cell bodies (Figure 4e). Since all Gap43 synapses are cholinergic (Meidinger, Hildebrandt‐Schoenfeld, & Illing, 2006), Gad65 synapses cannot be part of the process of synaptogenesis and must have been present before *SD*.

Under the electron microscope, Gap43(+) profiles were found close to Ncan‐rich PNNs by POD3, mostly failing to form contact with the neuron (Figure 6b). By contrast, morphologically mature presynaptic endings containing Gap43 were often attached to the plasma membrane toward POD7, now morphologically indistinguishable from Gad65(+) presynaptic boutons. Ncan has been described as growth impermissive (Asher et al., 2000; Friedlander, 1994; Katoh‐Semba, Matsuda, Watanabe, Maeda, & Oohira, 1998). Our observations are suggestive of an Ncan degradation in VCN as a precondition for synaptic maturation to occur.

### 5.5 Rebalancing of synaptic networks following *SD*

Confirming a previous study (Hildebrandt et al., 2011), we noted an *SD*‐related loss of glutamatergic endings in VCN, which is significant by POD3 and later (Figure 4j). Accompanying the loss of glutamatergic endings upon *SD*, the inhibitory synaptic contacts identified by gephyrin decline in number by 30%. However, the fraction of inhibitory synapses of all synaptic contact zones in VCN after *SD* dominates excitatory contacts (Hildebrandt et al., 2011). Here, we report a rise of Gad65 staining intensity on the affected side which we attribute to an upregulation of Gad65 in pre‐existing, stable synaptic contacts. The fact that Gad65(+) presynaptic endings seem embedded and stabilized by PNNs rich in Agg and transiently also in Ncan seems consistent with their metabolic rather than structural modulation.

### 5.6 Astrocytic MMP2 might degrade NCAN after *SD*

Synchronous to the post‐POD3 decline of Ncan staining in ipsilateral VCN, staining for the matrix metalloproteinase MMP2 rose, a change attributable to astrocytes (Fredrich & Illing, 2010; Fredrich et al., 2013). Astrocytic expression of MMPs is well documented in the literature (Ogier et al., 2006; Szklarczyk, Lapinska, Rylski, McKay, & Kaczmarek, 2002; Yin et al., 2006). An involvement of MMP2 in various regenerative processes after brain injuries has been reported (Verslegers, Lemmens, van Hove, & Moons, 2013). In vitro, MMP2 degrades Ncan, whereas equimolar amounts of, for example, MMP1 and MMP9 fail to do so (Turk, Huang, Piro, & Cantley, 2001). Implantation of olfactory ensheathing cells, a type of glia enwrapping nonmyelinated axons of the olfactory nerve, into the injured rat spinal cord leads to the degradation of Ncan in vivo through the secretion of MMP2 (Yui, Fujita, Chung, Morita, & Nishimura, 2014).

Upon unilateral *SD* of the auditory brainstem, regions rich in MMP2 locally correspond to regions rich in Gap43 (Fredrich & Illing, 2010). Moreover, changes of MMP2 expression after *SD* are conditional upon an effective re‐innervation revealed by Gap43 immunoreactivity (Fredrich & Illing, 2011). A release of MMP2 from astrocytes upon the approach of growing neurites and synaptogenesis locally and temporally coincides with the degradation of Ncan from PNNs in VCN.

### 5.7 AGG changes were moderate

Although Ncan and Agg are differently regulated in early ontogeny, they changed in a similar direction in our experimental model. Unlike Ncan, Agg is abundant in VCN of adult control brains, making Agg a widely present component of PNNs. Following *SD*, the expression of Agg in VCN changed only mildly but in the same vein as Ncan. This suggests an involvement of Agg in plastic processes as has been previously reported (Harris, Carmichael, Hovda, & Sutton, 2009; Lemons, Sandy, Anderson, & Howland, 2001; McRae, Rocco, Kelly, Brumberg, & Matthews, 2007; Ueno et al., 2018; Ye & Miao, 2013). As Ncan, Agg is susceptible to dismounting by MMP2 (Overall, 2002).

### 5.8 Putative functions of CSPGS in reorganization of the VCN

We suggest that Ncan and Agg are crucial components in the regulation of *SD*‐dependent re‐innervation in VCN. While several studies point to the growth impermissive properties of CSPGs (Asher et al., 2000; Friedlander, 1994; Katoh‐Semba et al., 1998), others suggest supporting effects of CSPGs on axon outgrowth (Haas et al., 1999; Maeda & Noda, 1996). Recently, it has been shown that the potential of CSPGs to support neuroplasticity depends on their sulfation pattern and decreases with age (Foscarin, Raha‐Chowdhury, Fawcett, & Kwok, 2017). In our experimental model, the rise and fall of Ncan and Agg might indicate an effort for emergency stabilization of synaptic contacts before neurite outgrowth triggers astrocytes to express and release MMP2 that degrades CSPGs in order to provide access for growth cones to form new synapses. Cutting efferent axons originating in the facial nucleus nerve led to glial scar formation but did not change levels of Ncan or Agg. In contrast to *SD* of the VCN, retrograde degeneration of the facial nerve did neither cause loss of synaptic input to the facial nucleus nor the formation of new synaptic contacts.

### 5.9 Astrocytes are key players in the *SD*‐dependent reorganization of VCN

Apart from their involvement in the management of tissue degeneration and glial scar formation, astrocytes also participate in recovery processes after brain lesions. Preventing astrocyte activation has negative effects on tissue reorganization. Unilateral cerebral stroke of the motor cortex in mice normally induces axons of the contralesional corticospinal tract to sprout into the denervated spinal cord, contributing to motor functional recovery (Liu, Li, Zhang, Savant‐Bhonsale, & Chopp, 2008). When astrogliosis is attenuated by double knockout of GFAP and vimentin in mice that then underwent stroke of motor cortex, corticospinal axons only rarely crossed the midline and their length was significantly reduced compared to the axons in wild‐type mice. The reduced astrocytic reactivity leads to impaired neurological recovery (Liu et al., 2014). In our *SD* model, astrocytes in VCN are activated, grow, branch, and express PSA‐NCAM, MMP2, and MMP9 (Fredrich et al., 2013). Their expression of PSA‐NCAM and MMP2 is conditional upon the arrival of Gap43(+) nerve fibers (Fredrich et al., 2013). Here, we show that, shortly after *SD*, they also synthetize Ncan and then engage in MMP2 expression. This succession suggests being causal to the degradation of Ncan through the released MMP2, thus providing access for neurites to form new synaptic contacts.

### 5.10 Transient transsynaptic reaction of the contralateral IC on POD1

By POD1, expression of Ncan, Agg, Gap43, and vGluT1 was found to be increased in the contralateral CIC that does not receive auditory‐born signals directly by cochlear axons. The quick responses were remarkable given their remoteness to degenerational processes. The molecular modulations seen in CIC were transient. Compared to VCN, changes were mild but significant. A quick but brief effect was the same for all molecular markers employed except for Gad65. The level of Gad65 staining decreased in contralateral CIC and remained low, indicating a lasting effect on network activity (Mossop, Wilson, Caspary, & Moore, 2000; Vale, Juíz, Moore, & Sanes, 2004). Taken together, fast molecular adaptations occurred in CIC as a consequence to cochlear lesioning, some transient and some sustained, which together indicate remarkable quick and specific transsynaptic modifications of signal processing in the auditory midbrain. In chinchillas, about 50% of collicular neurons significantly increase their spiking rate upon acoustic trauma, an  effect that the authors associate with loss of inhibition (Wang, Ding, & Salvi, 2002). This enhancement shows up as quickly as by 8h post‐trauma. Indeed, a short‐term rise of excitability in CIC neurons upon hearing loss has been demonstrated before (McAlpine, Martin, Mossop, & Moore, 1997; Popelár̆, Erre, Aran, & Cazals, 1994; Salvi, Saunders, Gratton, Arehole, & Powers, 1990; Szczepaniak & Møller, 1996; Willott & Lu, 1982). These electrophysiological studies give no clue about underlying molecular correlates. CSPGs were reported to protect cortical and hippocampal neurons from excitotoxicity (Okamoto, Mori, Ichimura, & Endo, 1994). In the rat hippocampus, neuronal hyperactivity induced by electrical stimulation induces astrocytic expression of Ncan (Schwarzacher et al., 2006). A protection of collicular neurons against overexcitation could be reflected by the growth of Ncan‐enriched PNNs as reported here.

## CONFLICT OF INTEREST

The authors disclose no conflict of interests.

## Data Availability

The materials used in this study are made fully explicit and are available from public suppliers. Brain sections, protocols, and measurement data on which this study is based are fully archived and accessible in the laboratory library of our department. Animals were obtained from Charles River Laboratories (https://www.criver.com, RRID: RGD_13508588). Chemicals used in this study were provided by Carl Roth (https://www.carlroth.com; Sudan Black B; Mowiol 4‐88; DABCO), Vector Laboratories (https://vectorlabs.com; Elite Vectastain ABC Kit), Sigma‐Aldrich (https://www.sigmaaldrich.com; DAB), Merck Millipore (http://www.merckmillipore.com; Entellan), Aurion (https://aurion.nl; BSA‐c), Nanoprobes (http://www.nanoprobes.com; GoldEnhance EM), and Science Services (https://www.scienceservices.de; Embed 812). Antibodies were purchased from Merck Millipore (http://www.merckmillipore.com), Sigma‐Aldrich (https://www.sigmaaldrich.com), Molecular Probes (https://www.thermofisher.com), Santa Cruz Biotechnology (https://www.scbt.com), R&D Systems (https://www.rndsystems.com), Vector Laboratories (https://vectorlabs.com), Abcam (https://www.abcam.com), and Nanoprobes (http://www.nanoprobes.com) (for RRIDs, see Table 1). For image acquisition and editing, we used AxioVision (https://www.zeiss.com, RRID: SCR_002677), Leica Application Suite X (https://www.leica-microsystems.com, RRID: SCR_013673), Huygens Deconvolution (https://svi.nl, RRID: SCR_014237), Fiji (https://fiji.sc, RRID: SCR_002285), Adobe Photoshop CS6 (https://www.adobe.com, RRID: SCR_014199), and Imaris 9.2.0 (https://imaris.oxinst.com, RRID: SCR_007370). Statistical analysis was performed with Prism 8.1.0 (https://www.graphpad.com; RRID: SCR_002798).
