# Supplementary figures and images for: Sensory deafferentation modulates and redistributes neurocan in the rat auditory brainstem
Source: Brain Behav. 2019 Jul 4;9(8):e01353. doi: 10.1002/brb3.1353 (PMC6710208; doi:10.1002/brb3.1353)

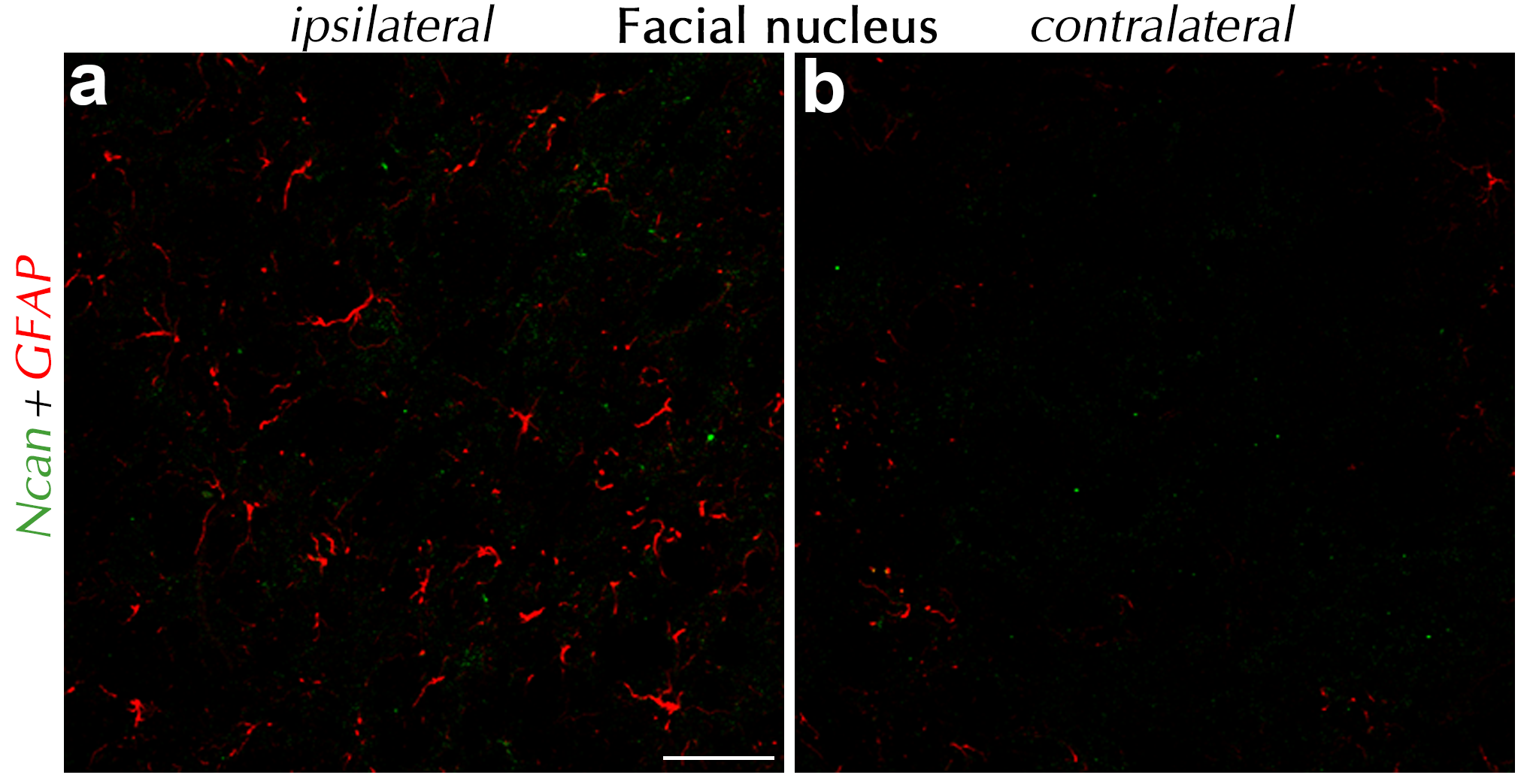

Supplement: Supplementary file 3 [file BRB3-9-e01353-s003.tif]
