# Supplementary material for: Sensory deafferentation modulates and redistributes neurocan in the rat auditory brainstem
Source: Brain Behav. 2019 Jul 4;9(8):e01353. doi: 10.1002/brb3.1353 (PMC6710208; doi:10.1002/brb3.1353)
Supplement: Supplementary file 4 [file BRB3-9-e01353-s004.docx]

**Supplementary 3 – Figure Legend**. Cutting the facial nerve during the process of cochleotomy causes a massive growth of GFAP(+) processes in the affected, ipsilateral facial nucleus (**a**) at POD3. No such effect was seen on the unaffected, contralateral side (**b**). Ncan expression was sparse in facial nucleus and remained unaltered despite facial nerve lesion. Scale bar: 50µm.
